# Complementary Feeding in Italy: From Tradition to Innovation

**DOI:** 10.3390/children8080638

**Published:** 2021-07-26

**Authors:** Patrizia Alvisi, Marco Congiu, Monica Ficara, Patrizia De Gregorio, Roberto Ghio, Enzo Spisni, Pietro Di Saverio, Flavio Labriola, Doriana Lacorte, Paolo Lionetti

**Affiliations:** 1 Pediatric Gastroenterology Unit, Maggiore Hospital, 40132 Bologna, Italy; flavio.labriola@ausl.bologna.it; 2 Residency School of Pediatrics, University of Bologna, 40138 Bologna, Italy; marc.congiu@gmail.com; 3 Pediatric Unit, Bufalini Hospital, 47521 Cesena, Italy; monica.ficara@auslromagna.it; 4 AUSL Teramo, 64100 Teramo, Italy; patrizia.degregorio@gmail.com (P.D.G.); pietro.disaverio@studiopediatrico.org (P.D.S.); 5 Independent Researcher, 50051 Castelfiorentino, Italy; roberto.ghio@gmail.com; 6 Department of Biological, Geological and Environmental Sciences, University of Bologna, 40126 Bologna, Italy; enzo.spisni@unibo.it; 7 Independent Researcher, 40100 Bologna, Italy; dorianalacorte@libero.it; 8 Pediatric Gastroenterology and Nutrition Unit, Meyer Children’s Hospital, 50139 Firenze, Italy; paolo.lionetti@unifi.it

**Keywords:** complementary feeding, weaning practice, baby-led weaning, personalized nutrition, Italian regions, food tastings

## Abstract

Complementary feeding (CF) is a pivotal phase of the individual’s growth, during which children develops their future dietary habits. To date, only few studies investigated and compared weaning modalities between different geographical areas. The aim of this article is to describe the current Italian practice for CF in healthy term infants among different areas (North, Center, South) of Italy. Two different multiple-choice questionnaires were produced and sent to 665 Italian primary care pediatricians (PCP) and 2023 families with children under 1 year of age. As emerged from our investigation, in Italy CF is usually started between the 5th and 6th month of life. The preferred approach (chosen by 77% of families) involves the use of home-cooked liquid or semi-liquid ailments, or industrial baby foods. A new CF modality is emerging, consisting of traditional complementary foods with adult food tastings (10% of families). Approximately 91% of pediatricians give written dietary suggestions, and 83% of families follow their advice. We found significantly divergent weaning habits among different areas of Italy. PCP have a key role in guiding parents during the introduction of new foods in their infant’s diet and should take this as an opportunity to educate the whole family to healthy dietary habits.

## 1. Introduction

Complementary feeding (CF) is a process that involves the gradual decrease in frequency and volume of milk intake (breast milk or formula) with the introduction in the infants’ diet of both solid and liquid ailments, namely complementary foods, which may be homemade with fresh ingredients, or they can be ready-made commercial baby foods (BFs) (industrially processed ailments belonging to group 4 of the NOVA classification) [1], suitable to be used as first foods for infants as they have a proper nutrients balance.

CF should be started between the 4th and 6th month of life. It becomes necessary since milk alone cannot guarantee a regular growth of the child beyond a certain age because of his/her increased nutritional requirements—most importantly iron needs [2]. "Weaning" can be used as a synonym for "complementary feeding", although the latter is preferable because it better recalls the very nature of this practice: introducing solid foods in the diet, while milk remains an important source of nourishment [3]. CF is a fundamental milestone in an infant’s growth. It takes place in a critical period of life, during which the interaction between the environment (including the nutritional model) and the individual genetic predisposition could play a role in developing medical conditions such as cardiovascular diseases, diabetes, and metabolic syndrome [4].

The Italian National Healthcare System provides all children with a family care pediatrician, who has an institutionalized role in caring for the pediatric population over time, focusing especially on their growth and overall health—including nutrition. In this context, parents’ most important task is to offer a wide variety of aliments: they should not be concerned about the quantity but rather the quality of foods on their tables. This also improves the children’s acceptance of new tastes, as in this phase they are willing to experience foods other than milk: CF should be regarded as an opportunity to educate them to heathy feeding habits based on the intake of various fresh foods.

Even though many recommendations have been made [2,5,6], there are substantial variations in CF modalities between European countries due to different traditions. Moreover, many families adopt new styles of CF, challenging the habits from the past decades. In this context, the shortage of evidence-based data on this topic creates confusion which translates into different behaviors among both pediatricians and families.

Currently, there are limited data about CF practices in Italy [7,8]. To our knowledge, no previous study compared the habits related to CF between Italian geographical areas. The aims of this paper are to describe the current CF practices in Italyto shed lighton the differences between different geographical areas.

## 2. Materials and Methods

We produced and submitted to Italian PCPs an ad-hoc multiple-choice questionnaire concerning their habits about CF. It was sent to pediatricians reached through the RePER (Rete Pediatrica di Epidemiologia e Ricerca), a network under the direction of the Pediatric Medical Italian Society, during a timespan ranging from January 2015 to December 2017. The questionnaire was filled out on a voluntary basis. The questionnaire consisted of 4 items regarding: (1) the method of proposed CF (sweet milk flour weaning, l spoon-feeding, self-weaning, traditional spoon-feeding supplemented with adult food tastings); (2) the suggested age of introduction of CF; (3) the habit of giving or not to parents written information; and (4) the pediatrician’s opinion about the use of baby foods (Table 1).

We produced and sent to Italian families an ad-hoc multiple-choice questionnaire investigating their attitude towards CF, during a timespan ranging from June 2018 to December 2018. The questionnaire was filled out on a voluntary basis. Families involved were chosen from the ones assigned to PCPs who were part of the RePER. The PCPs involved in this step of the analysis were part of a different pool than the ones who filled out the questionnaire as described above. We only included families with healthy full-term newborns. Children with congenital anomalies or any other medical issue (as food intolerance, allergies, metabolic diseases, etc.) were excluded because of the special needs of this group potentially influencing CF. The questionnaire consisted of 5 items regarding: (1) the initial method used for infant nutrition (breastfeeding, formula feeding, mixed); if breastfed, also the duration of breastfeeding was asked; (2) the timing of complementary foods introduction; (3) the method of CF used; (4) the use of baby food; and (5) the adherence to the indications provided by the pediatrician (Table 2). The questionnaire was designed to be easy to fill out, regardless of the educational of level of parents, and it was filled out retrospectively by one or both of the parents, indifferently.

Concerning the different styles of CF, we defined each method as follows:

- “Sweet milk flour”: a popular method used in the past decades, with sweet milk flour as the base for making the first weaning food, nowadays it has been almost completely abandoned.
- “Traditional spoon-feeding”: a widely used method involving feeding babies homemade liquid or semi-liquid foods, or commercial BFs. The basic plate in this case is made of vegetable broth with semolina or rice flour, freeze-dried meat or fresh meat, olive oil and parmesan cheese.
- “Traditional spoon-feeding with adult food tastings”: while following a traditional CF style, parents often decide to let their children have a tasting of other foods, especially when sharing the meal. In this case, parents mash and mince the food they are eating and feed it to their child, mixing the traditional approach with some aspects typical of other methods of CF (such as BLW or self-weaning).
- “Self-weaning”: an on-demand approach pioneered by the Italian pediatrician Lucio Piermarini [9], similar to the baby-led weaning (BLW) method described by Rapley [10] considering that the food offered to the infant is partly the same that the parents eat; the main difference is that in the self-weaning approach the food is minced, mashed and spoon-fed instead of being hand-held by the infant.

We did not consider formula milk as a complementary food, but rather as an equivalent of breast milk. Therefore, we considered CF to be started when children began to eat foods other than milk (both breast and formula).

**Figure 1:**
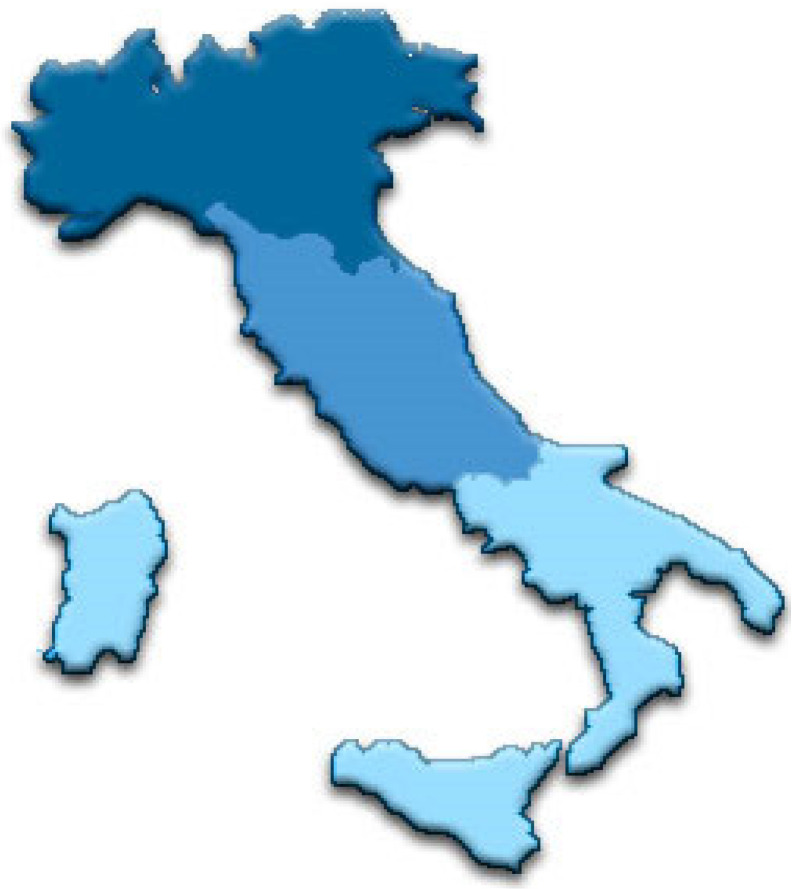
Division of Italy in geographical areas: North (dark blue), Center (blue) and South (light blue).

We identified and arbitrarily divided Italy in three geographical areas based on similarities from the socio-cultural perspective, as shown in Figure 1.

Categorical variables were summarized by the total number (n) and frequency (%). The association between categorical variables (2-way tables) was tested by the χ^2^ test. Statistical analysis was performed with STATA software (v. 15.1). *p* values <0.01 were considered significant.

## 3. Results

We collected a total of 665 filled out questionnaires from PCPs (95% of the PCPs respondents): 207 from Northern, 302 from Central and 156 from Southern regions of Italy. The questionnaire was filled out by approximately 9% of the PCPs working in Italy during the examined timespan (about 6.5% of the PCPs from Northern regions, 17% from the Center and 6% from the South of Italy) [11].

**Figure 2:**
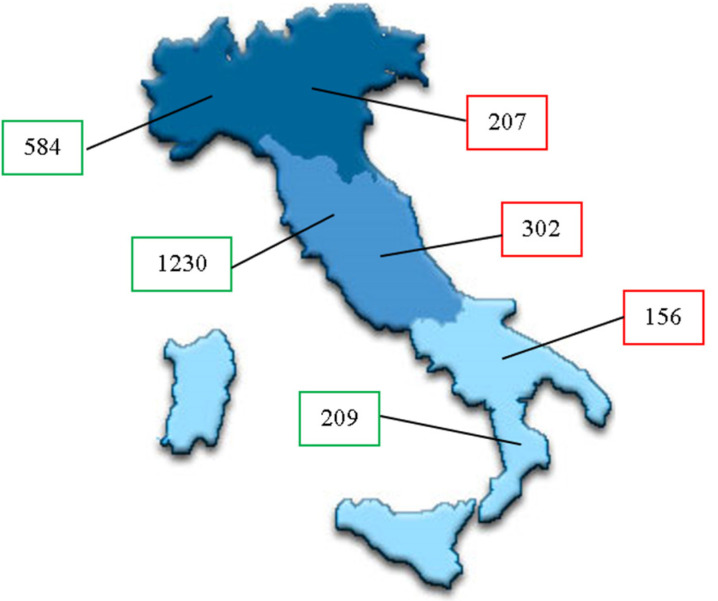
Distribution of PCPs (numbers showed on the right) and families (on the left) interviewed across Italian regions (North, Center and South).

The questionnaires collected from families were 2023: 584 from Northern regions, 1230 from Central and 209 from Southern ones (Figure 2).

### 3.1. Questionnaire to Pediatricians

#### 3.1.1. Type of CF

We report an overall rate of 60% (397/665) PCPs recommending the traditional spoon-feeding approach, mainly in the South (73% of Southern pediatricians) and Center (62%) of Italy; this practice is less common in the North (48%). On the other hand, 184 pediatricians (28%) suggest spoon-feeding with adult food tastings (36% of Northern pediatricians, 27% of Central and 17% of Southern ones). Only 82 (12%) follow the self-weaning approach (with similar percentages among different areas), and no one endorses sweet milk flour as an ailment suitable for CF.

#### 3.1.2. Age of CF Introduction

Overall, 605 of the interviewed pediatricians (90%) suggest starting CF between the 5th and 6th month of life, with a slight predilection for the 5th month (308/665, 46%). Only 50 (7.5%) propose the introduction of CF at 4 months of life and 10 (1.5%) beyond 6 months.

#### 3.1.3. Written Information

In total, 604(91%) provide families with written information about CF, with no statistical difference between the three areas.

#### 3.1.4. BFs

In total, 422 of the interviewed pediatricians (63%) endorse the use of BFs, mainly in Southern regions (*p* < 0.01).

The results are summarized in Table 3.

### 3.2. Questionnaire to Families

#### 3.2.1. Breastfeeding

In total, 1570 out of 2023 families filled out this item (the other items were filled out by all the participants). Our survey reports 1115 women (71%) breastfeeding their babies, and 455 Italian families (29%) using formula milk. Specifically, in Northern regions families resort to exclusive formula feeding to a lesser extent (17% of Northern families) and there is a greater rate of breastfeeding until 6 months (57%) as compared to Central (32% and 46% respectively) and Southern ones (27.7% and 40%) (*p* < 0.01 and *p* < 0.001).

#### 3.2.2. Timing of Complementary Foods Introduction

Most of the interviewed families (77%) start CF between the 5th and 6th month with a slight predilection for the 6th month (817/2023, 40%). Overall, 13% of families start CF before 5 months, (7.5% in the North, 16% in the Center and 15.3% in the South; *p* < 0.001). Conversely, 10% of families start CF after 6 months, with no significant differences between regions.

#### 3.2.3. Type of CF

The traditional spoon-feeding CF is preferred by 1555 families (77% of the total), mainly in Central (84%) and Southern (78,9%) regions than in the North of Italy (61%) (*p* < 0.001). Only 73 families (4% of the total) chose CF with milk flour. Self-weaning has a marginal role, being practiced only by 190 (9%) families involved in this analysis and it is more frequent among families living in Northern Italy (14%). However, 205 families (10%), while carrying on traditional spoon-feeding, allow their children to taste adult food during mealtime, especially in Northern regions (*p* < 0.001).

#### 3.2.4. BFs

As regards the type of food proposed, 974/2023 families (48%) report using both homemade complementary foods and commercial BFs. The exclusive use of BFs is more frequent among Northern (43%) families compared to the ones living in the Center (29%) and South (29%).

#### 3.2.5. Pediatrician Indications

In total, 1688 out of 2023 families (83%) adhere strictly to the indications given by PCPs. Only 74 families (4%) report not following the indications given at all. The adherence to pediatricians’ instructions is higher among the families living in the Southern (86%) and Central regions (87%) compared to Northern ones (76%).

The results are summarized in Table 4.

## 4. Discussion

As emerges from our data, in Southern regions pediatricians suggest introducing CF earlier, endorse an extensive use of BFs and apply more frequently the traditional spoon-feeding approach compared to the colleagues in Northern and Central regions of Italy. As regards parents’ habits, in Central and Southern regions we documented a shorter duration of exclusive breastfeeding, a greater propensity towards traditional spoon-feeding, and a greater compliance to PCPs’ advise; industrial products are used to a broader extent by Southern families.

### 4.1. Breastfeeding

The World Health Organization states that exclusive breastfeeding for the first 6 months of life, except specific conditions, is suitable to guarantee an adequate growth and regular development of the child [2,6]. It is to consider that breast milk composition varies according to the characteristics of the breastfed newborns (such as gestational age, postnatal age, number of breastfed babies), adapting to their specific needs [12,13]. In fact, exclusive breastfeeding up to 6 months efficiently supplies the nutritional requirements of children (except for vitamin D and K). Conversely, after 6 months of age, breastfeeding alone is no longer sufficient to provide an adequate intake of calories, iron, zinc, protein or fat-soluble vitamins.

According to our data, about 71% of women ever breastfed their babies, and 47% continued until 6 months of age. A significant number of families (23%) did not respond to this question, which could represent a bias in our analysis. We document less breastfed babies than reported by UNICEF in 2018 [14] and by the Italian National Institute of Statistics in 2014 [15]: according to these reports (which showed similar percentages), about 85% of children in Italy were breastfed, and exclusive breastfeeding was more common and more prolonged in the North [15]. Our data seem to confirm that ever-breastfed infants’ rates are lower in Central (68%) and Southern regions (72,3%) compared to the North of Italy (83%). Moreover, in the Center and South of Italy CF is started earlier than in the rest of the country, confirming the tendency already reported in literature, to begin CF earlier in formula-fed infants compared to breast-fed ones [16]. Among the factors affecting breastfeeding rate there is cesarean delivery, which is related to a lesser rate and duration of breastfeeding, and is indeed more common in Southern and Central regions [15]. Other factors reported affecting breastfeeding are: low maternal schooling and age, preterm birth, lack of adequate training given by health professionals, and persistence of old care strategies involving early feeding with infant formula [15,17].

### 4.2. Complementary Feeding

As regards the timing of CF, our report shows that most pediatricians and families (91% and 77%, respectively) begin CF between the 5th and 6th month of age. Only 8% of pediatricians suggest beginning CF at 4 months and the remaining 2% beyond the 6th month. The proper timing for beginning CF is a controversial topic of debate. It is a common opinion that defining a precise time for starting CF to be valid for all infants is not possible [14,18]. Some features hint at the infant’s readiness to start this process, such as the ability to sit unsupported, to grab objects with fine movements, bring them towards their mouth and chew them [19], and most importantly the interest shown towards foods in general [10].

The Italian Ministry of Health recommends CF to be routinely started after 6 months of life in healthy term babies, following the indications of the main scientific societies on this topic (WHO, European Society for Pediatric Gastroenterology Hepatology and Nutrition-ESPGHAN, European Food Society Authority-EFSA, American Academy of Pediatrics-AAP) [2,5,6]. If it were necessary to anticipate the beginning of CF (e.g., because of a significant deflection of the weight growth curve), it would be preferable to start it after the 4th month of life, when renal and gastrointestinal functions are mature enough and the child has acquired the motor skills needed [3]. Thus, there is no strict time frame for introducing new foods in the diet. However, CF should not be started before 17 weeks of life nor beyond 26 weeks: infants who begin CF out of this time frame are more likely to have bad feeding habits (e.g., they consume more unhealthy foods at one year of age) and to develop medical issues later in life, such as hypertension [20].

Delayed introduction of allergenic foods does not reduce the risk of developing food allergy, even if familiarity for atopy is present. Immunoregulatory and anti-inflammatory properties of allergenic foods could stimulate oral tolerance if introduced early in the infant’s diet [21,22,23]. Therefore, allergenic foods should be introduced into the diet between 4 and 6 months, the same way other foods are [24].

Parents have to offer their children a varied diet in terms of textures and tastes, including bitter vegetables according to season, to promote healthy feeding habits that respect their socio-cultural context. In this regard, BFs penalize the diversity of the various cuisine traditions, as commercial foods are similar to each other and rely mainly on the taste of sweet vegetables (such as potatoes and carrots). Throughout evolution, humans have developed an innate preference for sweet and salty tastes, associated to foods rich in energy and minerals. Such preferences are expressed even before birth and were useful in ancient environments where resources were scarce but have become a disadvantage in obesogenic contexts like our modern society is [2]. Breast and formula milk have a sweet taste, therefore weaning is an essential time to educate the infants to liking other tastes such as sour and bitter. While at birth the neonates show a preference towards sweetened solution versus plain water, at 6 months of life only infants who received routinely sweetened water during the first months maintained this preference [25]. This could be related to the fact that in this window of time (between 4 and 6 months), the receptors of non-sweet taste are fully active, and the child becomes attracted by non-milk foods [26]. This is only true if the preference for sweet taste has not been reinforced, and therefore the importance of letting the baby experience of a broad selection of foods.

According to the answers given to our questionnaires, the most well-established method for CF in Italy is the traditional spoon-feeding approach. This is recommended by 60% of PCPs, and only 13% of parents resorts to alternatives. The use of baby meals made with milk flour, widespread in the past decades, has been almost completely abandoned. The main innovation is that about 30% of pediatricians—while proposing the classic spoon-feeding as the core approach—invite parents to indulge the children’s curiosity, offering them some adult food tasting and the opportunity to take an active role during the family meal and experience a wider variety of flavors and textures. The early flavor experiences (including also the flavors perceived in the amniotic fluid and in breast milk) may provide the foundation for cultural and ethnic differences in dietary habits, because the infant is more likely to accept foods if they have already been known through maternal intake [27]. Moreover, the acceptance of new tastes could be influenced by genetics: there are genetic polymorphisms that regulate the perception of various tastes—especially bitter. This could explain why some infants are prone to try different foods and others have more selective eating habits [28]. The predilection for healthy food can be reinforced if the infant is constantly exposed to it. It is proven that repeated early exposure to the taste of some vegetables enhances the liking for them, and this effect on food acceptance persists up to 6 years later [2,29,30]. Some children may require 8–10 exposures before accepting one new flavor [31]. It is always important to introduce children to different flavors by repeating the exposure even after an initial refusal.

Self-weaning still seems to have a marginal role in Italy, being suggested only by 12% of interviewed pediatricians and chosen by 9% of families. Probably this result may be explained by parents’ fears such as the increased risk of choking, and the insufficient intake of iron (as well as other micronutrients) and energy. Regarding the risk of choking, evidence suggest that it is not increased in infants weaned with BLW compared to the traditional method [32]. As regards energy intake, no difference was observed between either [33]. Applying a structured approach to BLW (consisting in addressing the parents’ concerns before starting the CF), no significant differences were observed in terms of iron and zinc intake between BLW and traditionally weaned infants [34,35,36], although fibers intake was still lower in the BLW group, and saturated fat and sodium intake were higher [37].

From our investigation emerges that 63% of the interviewed pediatricians endorse the use of BFs, and commercial products (as the sole source of feeding or together with home-cooked foods) are used by 81% of families, mostly in Southern regions. This attitude could be explained by both the parents’ concern about the presence of contaminants in fresh foods, and the convenience in buying ready-made food. Noteworthy, the exclusive use of BFs seems to be more prevalent among Northern families. This could relate to an increasing trend in the Western society, where both parents usually work and have less time to cook. The nutritional adequacy of commercial products and home-cooked ailments is similar, even though the former have a more appropriate energy density and a lower content in fat [38]. The variety of vegetables in the meal seems to be poor both for commercial and homemade foods. This could be due to an innate preference for naturally sweet-tasting vegetables, which are the most used because readily accepted by the infant. This could impair the acceptance of some vegetables, especially bitter ones, whose flavor is masked by others. Commercial products present a slightly wider variety of ingredients per-meal, but homemade meals guarantee a greater diversification of the vegetables across various meals [38,39]. Nobleet al. found differences in weaning practice based on the type of milk received during the first months: formula-fed infants were less likely to appreciate fruits and vegetables and received more BFs compared to those breastfed [40]. Our study comes to similar conclusions considering that in Southern regions, where a lower number of breast-fed babies is observed, there is a wider use of BFs. Among other factors influencing CF, a study conducted in France reported that low socio-economic status (e.g., poor schooling and unemployment) is associated with a lesser adherence to French CF guidelines [41]. This could also explain the differences found in our survey between North and the other regions, considering that in the Center and South of Italy CF is started earlier and flexible feeding styles such as self-weaning or adult food tastings are less common than in the North.

Parental feeding style during CF can influence the offspring’s futures dietary habits and life-long feeding behavior [42]. Many families carry on inappropriate habits potentially increasing childhood obesity, such as feeding in response to the babies’ discomfort, or encouraging them to finish the food in the plate. In contrast, Komninou et al. found that parents who carried on BLW had less control over feeding and shared more time with their children during meals. Infants weaned with a spoon-feeding approach seemed to have a more selective diet and to be less amused during their meal [43]. Knowing and respecting the children’s signals suggesting they have had enough to drink and eat is thus considered the best approach. This attitude, referred as “responsive feeding” could help reduce the overnutrition and the resulting obesity, if applied during the first months of life [44].

### 4.3. Role of PCPs

Approximately 91% of interviewed PCPs provide a written schedule for CF for parents to follow. Pediatricians who have this habit use their own self-made forms, according to their personal experience and preference, as there is not a standardized model. Such schedules often contain very precise and rigorous information which may increase parents’ concern. Nevertheless, their advice is followed by most families, especially in the Central and Southern regions of Italy, where self-weaning is not common. This statement is consistent with previous findings, as parents applying the BLW approach followed pediatricians’ indications to a lesser extent [43]. Brambilla et al. conducted a study on a group of Italian pediatricians to define which criteria they rely on for starting CF. Most pediatricians reported starting CF between 5 and 6 months of age with different modalities (BLW or pre-arranged schedules) depending on the individual case and indicated the infants’ development as the main parameter for beginning CF rather than nutritional needs. Even from this report it is evident that an approach led by the baby is still not widely diffused and used mostly in the North of Italy; these findings are consistent with the results of our study [45]. Our data are also consistent with the results of a similar survey conducted in Spain. Spanish PCPs were reported to give written information to parents in 95% of cases. They mostly recommend traditional spoon-feeding and showed scarce tendency to the routine use of the BLW approach [46].

## 5. Conclusions

CF can be considered a period of growth and nutritional education for all the family members. Its medicalization should be avoided, as feeding restrictions are not supported by scientific evidence. On the contrary, they may limit infants’ curiosity and interests. PCPs should act as a guide for parents and help them to recognize infants’ signals suggesting their readiness to be weaned, especially their interest in food. Pediatricians’ indications are tools for the caregivers to ensure a nutritionally balanced diet, but excessively strict written indications about the type of food and the timing to introduce it seldom are of any benefit for both the children and their families.

In our opinion, a flexible approach that includes both spoon-fed pureed foods and some soft food deemed suitable by the family, may be a reasonable synthesis of the different weaning modalities currently in use in Italy. This synthesis of BLW and traditional CF, probably spontaneously adopted by many families, allows the child to benefit both from the positive implications of meal sharing and of a nutritionally adequate meal. It is necessary to satisfy infants’ demands, not to pose any constraint, and to offer fresh foods such as vegetables and fruits according to the season to develop a healthy feeding style. Indeed, feeding style learnt during infancy influences life-long dietary habits. Therefore, children’s interests should be supported, respecting the cultural and ethnic background to which they belong, and parents should be encouraged to offer a wide variety of foods even if the infant initially refuses them. Nowadays the primary care pediatrician is an important point of reference to improve eating style, and this opportunity should be used to intervene on the feeding style of the entire family. Accompanying parents in this path of food education, pediatricians help to prevent the development of chronic diseases later in life, such as diabetes mellitus, obesity, and arterial hypertension.

The meal is a time of sensory-motor exploration involving infants’ touch, sight, taste, and smell. It is up to pediatricians to teach families that weaning should be experienced as a time of discovery rather than a clash between parents and their children.

Our study has a few limitations. Firstly, we did not investigate the socio-cultural context of the families interviewed; this could have helped explain some of the differences pointed out by our survey. Secondly, the PCPs and families were not equally distributed across Italy, with the Center having a larger sample; this could have represented a bias in our analysis. Thirdly, we only considered families with healthy full-term infants with no history of medical conditions; therefore, our results cannot be generalized to the entire Italian pediatric population.

The major strength of our study is that, to the best of our knowledge, this is the first of its kind investigating the habits about CF in different Italian areas. We collected responses from a considerable number of PCPs (approximately 9% of Italian pediatricians), supporting the reliability of our investigation.

Further studies are needed to point out the reasons behind the differences we found and correlate them to specific risk factors for the Italian population.

## Figures and Tables

**Table 1:** Multiple-choice questionnaire sent to pediatricians.

**What style of CF do you usually suggest?**			
Sweet milk flour	Traditional spoon-feeding	Self-weaning	Traditional with adult food tastings
**What age do you suggest to begin CF at?**			
4 months (91–120 days)	5 months (121–150 days)	6 months (151–180 days)	7 months (181–210 days)
**Do you provide families with written information about CF?**			
	yes	no	
**Do you endorse the use of commercial BFs for CF?**			
	yes	no	

**Table 2:** Multiple-choice questionnaire sent to families.

**Since Birth, Your Infant Has Been Fed with…**						
**Breast and Formula Milk**	**Only Formula Milk**		**Breast Milk until 3 Months**		**Breast Milk until 6 Months**	
When did you offer your infant complementary foods? (Months of age)						
3	4	5	6	7	8	>8
What style of CF did you use?						
Sweet milk flour	Traditional spoon-feeding		Self-weaning	Traditional with adult food tastings		
Did you feed your infant commercial BFs?						
yes		no		sometimes		
Did you follow your family care pediatrician’s indications?						
yes		no		partly		

**Table 3:** Answers of care primary pediatricians and differences based on the area of origin.

Questionnaire Items	Italy	North	Center	South	*p* Value
	*n* = 665	*n* = 207	*n* = 302	*n* = 156	
Style of CF * n (%)					
Traditional spoon-feeding	397 (60%)	99 (48%)	185 (62%)	113 (73%)	**<0.01**
Self-weaning	82 (12%)	33 (16%)	34 (11%)	15 (10%)	0.14
Traditional with adult food tastings	186 (28%)	75 (36%)	82 (27%)	27 (17%)	**<0.01**
Age (months) *n* (%)					
4	50 (7.5%)	7 (3%)	21 (7%)	22 (14%)	**<0.01**
5	308 (46%)	107 (52%)	122 (41%)	79 (51%)	0.02
6	297 (45%)	91 (44%)	155 (51%)	51 (33%)	**<0.0001**
7	10 (1.5%)	2 (1%)	4 (1%)	4 (2%)	0.41
Written information *n* (%)					
yes	604 (91%)	185 (89%)	283 (94%)	136 (87%)	0.049
no	61 (9%)	22 (11%)	19 (6%)	20 (13%)	0.049
Advise of BFs *n* (%)					
yes	422 (63%)	125 (60%)	171 (57%)	126 (81%)	**<0.01**
no	243 (37%)	82 (40%)	131 (43%)	30 (19%)	**<0.001**

Bold indicates most relevant *p* values. * CF with sweet milk flour was suggested by none of the interviewed pediatricians.

**Table 4:** Data collected from families and differences based on the area of origin.

Questionnaire Items	Italy	North	Center	South	*p* Value
	*n* = 2023	*n* = 584	*n* = 1230	*n* = 209	
Type of milk * n (%)					
Mixed	248 (16%)	45 (16%)	175 (15%)	28 (21.5%)	0.144
Only artificial	455 (29%)	46 (17%)	373 (32%)	36 (27.7%)	0.011
Maternal until 3 months	126 (8%)	26 (10%)	86 (7%)	14 (10.8%)	0.242
Maternal until 6 months	741 (47%)	156 (57%)	533 (46%)	52 (40%)	
Style of CF *n* (%)					
Sweet milk flour	73 (4%)	27 (5%)	33 (3%)	13 (6.2%)	0.01
Traditional spoon-feeding	1555 (77%)	359 (61%)	1031 (84%)	165 (78.9%)	**<0.001**
Self-weaning	190 (9%)	79 (14%)	91 (7%)	20 (9.6%)	**<0.001**
Traditional with adult food tastings	205 (10%)	119 (20%)	75 (6%)	11 (5.3%)	**<0.001**
Age (months) *n* (%)					
3	23 (1%)	4 (0.7%)	16 (1%)	3 (1.4%)	0.467
4	248 (12%)	40 (6.8%)	179 (15%)	29 (13.9%)	**<0.001**
5	740 (37%)	230 (39.7%)	428 (35%)	80 (38.3%)	0.041
6	817 (40%)	249 (44%)	482 (39%)	79 (37.8%)	0.122
7	103 (5%)	28 (4.8%)	65 (5%)	10 (4.8%)	0.886
8	32 (2%)	10 (1.7%)	19 (2%)	3 (1.4%)	0.950
>8	60 (3%)	14 (2.3%)	41 (3%)	5 (2.4%)	0.479
Use of BFs *n* (%)					
yes	667 (33%)	253 (43%)	353 (29%)	61 (29%)	**<0.001**
no	382 (19%)	118 (20%)	244 (20%)	20 (10%)	0.01
sometimes	974 (48%)	213 (37%)	633 (51%)	128 (61%)	**0.001**
Adherence to indications *n* (%)					
yes	1688 (83%)	445 (76%)	1063 (87%)	180 (86%)	**<0.001**
no	74 (4%)	19 (3%)	54 (4%)	1 (1%)	0.017
partly	261 (13%)	120 (21%)	113 (9%)	28 (13%)	**<0.001**

Bold indicates most relevant *p*-values. * 1570/2021 families answered this question; for this item we considered 1570 as the total to calculate percentage distribution.

## Data Availability

The data presented in this study are available on request from the corresponding author.
